# Type 2 Diabetes Susceptibility Gene Expression in Normal or Diabetic Sorted Human Alpha and Beta Cells: Correlations with Age or BMI of Islet Donors

**DOI:** 10.1371/journal.pone.0011053

**Published:** 2010-06-10

**Authors:** Clare L. Kirkpatrick, Piero Marchetti, Francesco Purrello, Salvatore Piro, Marco Bugliani, Domenico Bosco, Eelco J. P. de Koning, Marten A. Engelse, Julie Kerr-Conte, François Pattou, Claes B. Wollheim

**Affiliations:** 1 Department of Cell Physiology and Metabolism, University of Geneva, Geneva, Switzerland; 2 Metabolic Unit, Department of Endocrinology and Metabolism, Cisanello Hospital, Pisa, Italy; 3 Department of Internal Medicine, Garibaldi-Nesima Hospital, University of Catania, Catania, Italy; 4 Cell Isolation and Transplantation Centre, University Hospital, Geneva, Switzerland; 5 Department of Nephrology, Leiden University Medical Centre, Leiden, The Netherlands; 6 INSERM U859, University Lille Nord de France, University Hospital of Lille, Lille, France; University of Bremen, Germany

## Abstract

**Background:**

Genome-wide association studies have identified susceptibility genes for development of type 2 diabetes. We aimed to examine whether a subset of these (comprising *FTO*, *IDE*, *KCNJ11*, *PPARG* and *TCF7L2*) were transcriptionally restricted to or enriched in human beta cells by sorting islet cells into alpha and beta – specific fractions. We also aimed to correlate expression of these transcripts in both alpha and beta cell types with phenotypic traits of the islet donors and to compare diabetic and non-diabetic cells.

**Methodology/Principal Findings:**

Islet cells were sorted using a previously published method and RNA was extracted, reverse transcribed and used as the template for quantitative PCR. Sorted cells were also analysed for insulin and glucagon immunostaining and insulin secretion from the beta cells as well as insulin, glucagon and GLP-1 content. All five genes were expressed in both alpha and beta cells, with significant enrichment of *KCNJ11* in the beta cells and of *TCF7L2* in the alpha cells. The ratio of *KCNJ11* in beta to alpha cells was negatively correlated with BMI, while *KCNJ11* expression in alpha cells was negatively correlated with age but not associated with BMI. Beta cell expression of glucagon, *TCF7L2* and *IDE* was increased in cells from islets that had spent more time in culture prior to cell sorting. In beta cells, *KCNJ11*, *FTO* and insulin were positively correlated with each other. Diabetic alpha and beta cells had decreased expression of insulin, glucagon and *FTO*.

**Conclusions/Significance:**

This study has identified novel patterns of expression of type 2 diabetes susceptibility genes within sorted islet cells and suggested interactions of gene expression with age or BMI of the islet donors. However, expression of these genes in islets is less associated with BMI than has been found for other tissues.

## Introduction

Type 2 diabetes, with the exception of rare monogenic forms of the disease [1], arises from interactions between the genetic background of the patient and their environment. While the environmental component, for example the impact of obesity, has been well studied [2], examining the impact of genetic background on this disease has been more difficult. The most powerful studies in this field have been genome-wide association studies seeking to correlate single nucleotide polymorphisms (SNPs) with incidence of diabetes in very large sample sizes [3], [4], [5]. These studies have uncovered or confirmed several candidates for genetic determinants of type 2 diabetes susceptibility. Subsequent to these findings, other studies have sought to associate either SNPs in these genes, or expression of their RNA, with phenotypic characteristics of tissue donors. However, these have often focused on peripheral tissues such as adipose tissue, liver and muscle. Despite the importance of beta cell function to the development of diabetes, less attention has been given to the expression of these genes in human pancreatic islets (with the exceptions of *KCNJ11* and *TCF7L2*) due to the lesser availability of this tissue. In the present study we examined expression of *FTO*, *IDE*, *KCNJ11*, *PPARG* and *TCF7L2* in sorted human islet cells. These genes were among the first type 2 diabetes susceptibility genes to be identified and are some of the most well characterised with regard to their function (although it should be noted that the association is between diabetes risk and SNPs, which are not necessarily related to functional differences in the gene, and for *IDE* it is as yet unclear if the risk SNP is located in the *IDE* or the adjacent HHEX locus). In the case of *KCNJ11* and *TCF7L2* we wished to investigate the relative distribution of these transcripts between alpha and beta cells, while for *FTO*, *PPARG* and *IDE* we aimed to find whether these genes are transcribed in islet cells at all. In all cases, we were interested to see whether expression correlated with age or BMI. We also examined insulin and glucagon mRNA expression, as positive controls for each cell type.

In peripheral tissue, correlations have been observed between the BMI of the tissue donors and mRNA expression of type 2 diabetes susceptibility genes including *FTO*, *PPARG* and *TCF7L2* [6], [7], [8], [9], [10], [11]. Interestingly, high levels of FTO mRNA were not only associated with obesity but also with high levels of mRNA expression of the adipokines leptin, perilipin and visfatin [11]. In islets, *TCF7L2* has been extensively studied, but generally by analyzing the effect of SNPs on islet function, donor phenotype or probability of developing diabetes. Studies manipulating *TCF7L2* expression in islets have yielded mixed results, with some showing that increasing *TCF7L2* decreases glucose-induced insulin secretion and islet viability [12], while others show the opposite [13], [14]. Interestingly, Lyssenko and colleagues showed that there was a positive correlation between insulin mRNA and *TCF7L2* mRNA in human islets, although increasing *TCF7L2* mRNA also correlated negatively with glucose-stimulated insulin secretion [12]. However, recent work has suggested that *TCF7L2* mRNA and protein are oppositely regulated [15], so interpretation of results only examining *TCF7L2* mRNA should be treated with caution. Moreover, several splice variants of *TCF7L2* mRNA have been identified, with differing patterns of tissue-specific expression including one variant that is unique to pancreatic islets [16]. *KCNJ11* has been known to be a component of the ATP-sensitive potassium channel for many years, with clear implications for both insulin and glucagon secretion. Heterozygous activating point mutations in the protein have been identified in cases of permanent neonatal diabetes [17], in addition to the SNPs associated with mildly increased risk of type 2 diabetes [18], [19], [20]. However, its expression patterns within the human islet are not known, although it has been shown to be enriched in alpha cells in rat islets [21]. Less is known about the function of *IDE* in islets, although studies in the INS cell line have shown that it is capable of degrading amylin as well as insulin, and that its amylin-degrading function may be important for the function of the beta cell by keeping it clear of amyloid-like aggregates [22], [23]. An *IDE* knockout mouse exhibited hyperinsulinism and glucose intolerance, thought to be due to insufficient insulin clearance by the liver [24].

In this study we sorted human islet cells into beta and alpha fractions using a FACS-based method [25]. This has previously been used for investigating mRNA expression in purified beta cells [26]. It also possesses the advantage of providing both alpha and beta cell types, and with higher yield than can be obtained with laser capture microdissection [27]. Since type 2 diabetes is a bihormonal disease with dysregulation of alpha cell as well as beta cell function, this method is especially valuable since each cell type can be analysed in isolation. We hypothesised that genes which are known to be expressed in islets and which confer susceptibility to type 2 diabetes could display enrichment in the beta cells, or correlate with phenotypic characteristics of the islet donors, as has been found in other tissues [6], [7], [8], [9], [10], [11]. We therefore aimed to examine the expression of the selected type 2 diabetes susceptibility genes in both fractions and search for correlations between gene expression in either fraction, or the ratio of expression between the alpha and beta cells, with the age and BMI of the islet donors. In this context, *PPARG* should act as a negative control since it is thought to exert its influence on type 2 diabetes susceptibility through its action in other tissues (adipose tissue and liver) rather than the islet. Finding such correlations between islet cell-specific mRNA expression of type 2 diabetes susceptibility genes and phenotypic characteristics of the islet donors, or differences in gene expression between diabetic and non-diabetic islet cells, could therefore shed light on novel mechanisms of regulation of alpha or beta cell function. This study thus adds to the body of knowledge on the tissue-specific expression of these important genes and indicates further experimental directions to follow with regard to their effect on islet cell function.

## Methods

### Ethics Statement

Human pancreata were harvested from brain-dead organ donors after informed consent was obtained in writing from family members. Each islet isolation centre (University of Pisa, Geneva University Hospital, Leiden University Medical Centre and the University Hospital of Lille) had permission to isolate islets and to use them for scientific research if they are insufficient for clinical islet transplantation, in accordance with national laws and institutional ethical requirements. Ethical approval for this project was given by the Central Institutional Review Board on Clinical Research of Geneva University Hospital.

### Islet and cell culture

Intact human islets and sorted islet cells were cultured in CMRL 1066 medium (5.6 mM glucose) supplemented with 10% FCS, 1 mM Hepes, 100 U/ml penicillin, 100 µg/ml streptomycin and 100 µg/ml gentamicin at a density of approximately 10,000 IEQ per 20 ml medium. Islets were maintained in non-adherent 75 cm^2^ cell culture flasks (Corning) for at least one day before beta cell sorting to allow recovery from the stress of the islet isolation procedure and to expose all islets to uniform culture conditions. Details of islet donors are given in Table 1.

**Table 1:** Islet donor characteristics.

Diabetes status	Sex	n	Age (years)	BMI (kg/m^2^)
Non-diabetic	Male	8	46.75±8.61	26.79±4.34
Non-diabetic	Female	8	49.88±19.87	28.88±6.49
Type 2 diabetic	Male	3	52.33±6.17	28.27±1.39

Age and BMI are expressed as mean ± standard deviation. The SD of the age of the female islet donors is much larger than that of the male islet donors because the oldest and youngest islet donors were female; however, this did not affect the mean age.

### Beta cell purification

Dissociation of islet cells was achieved by incubation with constant agitation for 3 minutes at 37°C in 0.05% trypsin-EDTA (Invitrogen) supplemented with 3 mg/ml DNAse I (Roche) followed by pipetting vigorously to complete the dissociation. Labeling and sorting of alpha and beta cell fractions was performed as described [25] by Newport Green labeling [28] followed by FACS. Approximately 10,000 IEQ were used per isolation.

### Immunofluorescence

Sorted cells were spun down onto microscope slides by Cytospin, followed by fixation with 4% paraformaldehyde and washing in PBS. Cells were permeabilised in PBS +0.2% Triton X-100 for 15 minutes followed by blocking in PBS +0.5% BSA +0.05% Triton X-100 for 30 minutes. Cell spots were incubated with guinea pig anti-insulin and mouse anti-glucagon primary antibodies (diluted 1/200 in PBS), washed, and incubated with anti-guinea pig Alexa Fluor 568 and anti-mouse Alexa Fluor 488 (diluted 1/200 in PBS). Nuclei were stained with DAPI. The cells were examined by fluorescence microscopy and the percentage of insulin- and glucagon-positive cells in each fraction was quantified.

### Insulin secretion static incubations

This was performed as described [25] with cells seeded at a density of 20′000 cells per well in a 24-well plate, except that cells were also incubated with 5.6 mM glucose and with 20 mM KCl (in addition to 2.8 and 22.2 mM glucose). Insulin secretion and content were determined with an enzyme-linked immunoassay kit (SPI Bio).

### Glucagon and GLP-1 quantification

Acid ethanol extracts from beta cells, in which insulin content had already been measured, were used for glucagon and GLP-1 measurements. Glucagon was quantified using a ^125^I-labelled glucagon radioimmunoassay kit, while GLP-1 was quantified using an ELISA kit specific for the biologically active 7-36 amide and 7-37 forms of GLP-1 (both from Millipore). Quantification was by interpolation from standard curves prepared with known concentrations of glucagon or GLP-1, as appropriate. Values for each islet isolation were calculated from 12 replicates of 20′000 cells each, extracted in 0.3 ml of 5% acetic acid in ethanol at −20°C.

### Quantitative RT-PCR

RNA from sorted cells (100′000-500′000) was extracted with the Qiagen RNEasy Micro kit including an on-column DNAse I digestion step. RNA was converted to cDNA using SuperScript II (Invitrogen). Primers were designed using Primer Express software (Applied Biosystems). Primer sequences are provided in Table 2. Real time PCR was carried out on an ABI 7000 Sequence Detection system (Applied Biosystems) with the SYBR Green reagent using the standard curve method. Standard curve cDNA was prepared from total RNA of whole islets. Amplifications were performed in duplicate for all samples, and values normalised to the housekeeping gene RPS29.

**Table 2:** Quantitative PCR primer sequences.

Gene	NCBI accession no. of source	5′ primer3′ primer
RPS29	NM 001032	GAGCCACCCGCGAAAAT CCGTGCCGGTTTGAACAG
Insulin	BT 007778	GCAGCCTTTGTGAACCAACA TTCCCCGCACACTAGGTAGAGA
Glucagon	NM 002054	CAGGCAGACCCACTCAGTGA CTGGTGAATGTGCCCTGTGA
*FTO*	NM 001080432	GTTCACGGCCTGCACTCAT CCCTGGCTGCTTTGCTTAAT
*IDE*	NM 004969	CTCGGAACCTTGCTTCAACAC GGCCCGCTGAAGACGAT
*KCNJ11*	NM 000525	TTGGCAACACCGTCAAAGTG GAGGCGAGGGTCAGAGCTT
*PPARG*	NM 138712	GGCTTCATGACAAGGGAGTTTC AAACTCAAACTTGGGCTCCATAAA
*TCF7L2*	NM 001146274	CAGATGAAATGGCCACTGCTT GCATCCTTGAGGGCTTGTCTA

### Statistical analysis

Averaged data are displayed as mean ± SEM unless otherwise stated for the indicated n number (islet isolations from n donors). Significance of differences in averaged gene expression data between cell fractions were calculated by unpaired T test assuming equal variance. Spearman correlation coefficients, correlation p values and multivariate ANOVA were calculated using the program SPSS Statistics 17.0 (SPSS, Chicago, USA). Correlations were corrected for multiple testing by the Benjamini-Hochberg false discovery rate (FDR) method [29] with the FDR set at 5%. A value of p<0.05 after FDR correction was considered significant.

## Results

### Confirmation of cell fraction purity and function

**Figure 1:**
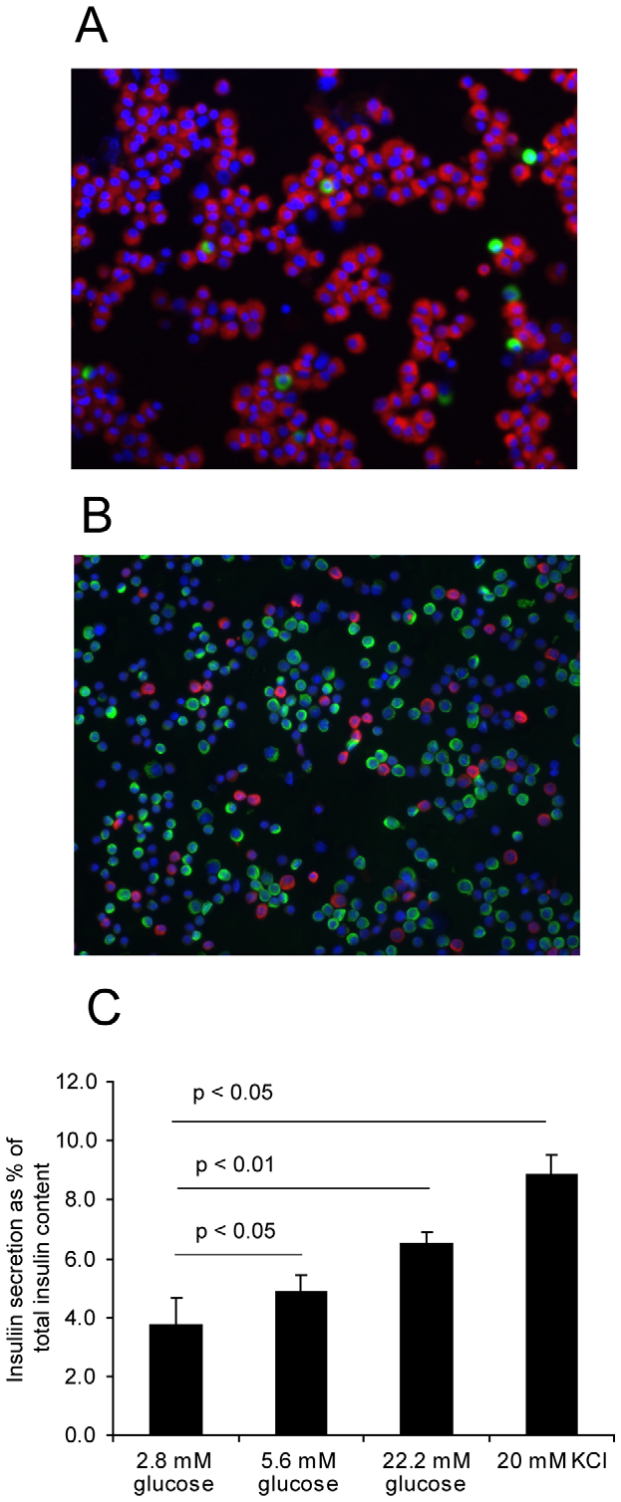
Characterization of alpha and beta cell fractions. A: Representative image of the beta cell fraction stained for insulin (red) and glucagon (green). B: Representative image of the alpha cell fraction stained likewise. C: Insulin secretion from sorted beta cells. Data are displayed as mean + SEM (islets from separate donors, n = 6 for all glucose concentrations and 5 for KCl). Statistical significance was assessed by one-tailed paired Student's T test comparing stimulated to basal secretion each time.

Sorted alpha and beta cells were applied to microscope slides and co-immunostained for insulin and glucagon in order to detect the amount of alpha cells in the beta cell fraction, and vice versa (Figure 1A and 1B, Table 3). We consistently obtained high purity (89%) beta cells, but the alpha cell fraction had a higher proportion of insulin and glucagon double negative cells (75% glucagon positive, 19% double negative). We found 6.2% insulin positive cells in the alpha cell fraction and 4.7% glucagon positive cells in the beta cell fraction, confirming that this sorting method efficiently separates the cell types. The insulin secretory response of the beta cells to glucose and potassium was also tested (Figure 1C). Consistent with previous data [25], we found that these cells responded to high glucose (22.2 mM) and potassium chloride (20 mM), although there was some variability between donors at basal glucose. This suggests that, at least in the six samples tested for insulin secretion, the beta cells have remained healthy despite the stress of islet isolation followed by cell sorting. To try to account for the variability between samples, we tested for correlations between insulin secretion at low glucose, and age, BMI or islet culture time. No association between any of these variables was found, so at present we cannot account for the source of this variability.

**Table 3:** Quantification of insulin and glucagon positive cells.

Cell fraction	Alpha	Beta
Insulin positive (%)	6.20±5.12	88.55±8.76
Glucagon positive (%)	74.47±11.12	4.73±3.31
Double negative (%)	19.33±6.63	6.73±6.34

Data are expressed as mean ± standard deviation of four independent experiments, with at least 800 cells counted per cell fraction for each one.

### Control gene profiling of sorted islet cell RNA

**Figure 2:**
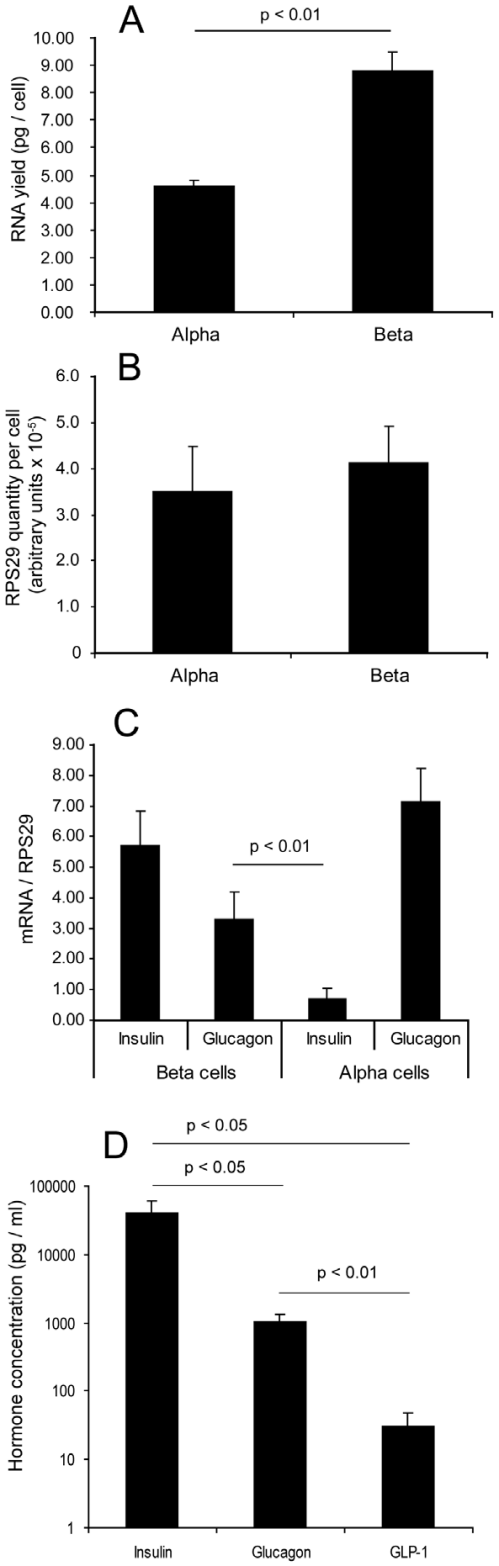
Control gene expression in sorted islet cells. A: RNA yield (picograms per cell) from the sorted islet cell fractions, expressed as mean + SEM (n = 4). B: Housekeeping gene RPS29 transcript quantity per cell, mean + SEM of quantitative RT-PCR measurements normalised to cell number (n = 6). C: Insulin and glucagon mRNA expression in both cell fractions, normalised to RPS29, mean + SEM from all non-diabetic islet donors (n = 16). D: Insulin, glucagon and GLP-1 content of beta cell fractions expressed in pg/ml (note logarithmic axis), mean + SEM of 6 non-diabetic islet isolations which were also used for insulin secretion measurements in Figure 1C. Statistical significance of differences in hormone concentrations was by Student's T test assuming equal variance.

RNA concentrations were measured from samples of sorted cells where the exact number of cells was known, in order to calculate the yield of RNA per cell (Figure 2A). It was found that the beta cells had almost twice as much RNA per cell as the alpha cells (4.58±0.22 pg/cell in the alpha cells but 8.77±0.70 pg/cell in the beta cells, p<0.01), consistent with their larger size. It was therefore necessary to confirm that the control gene for quantitative PCR was not over-represented in beta cells. We measured transcription of the control gene RPS29 (ribosomal protein subunit 29) in cDNA prepared from these samples, quantified it using the standard curve method and divided by the number of cells from which the RNA was prepared. There was no significant difference in RPS29 transcript quantity per cell between the cell fractions (Figure 2B), suggesting that beta cells do not overproduce this transcript relative to alpha cells. All values for both cell fractions were within two standard deviations of the mean, suggesting that there is also no significant difference in RPS29 transcript quantity per cell between islet donors. This result confirmed that RPS29 is an appropriate control gene for quantitative PCR in this context and that it will not introduce bias when comparing relative amounts of other transcripts between the alpha and beta cells. Having established that we had an appropriate control gene, we measured insulin and glucagon transcript levels normalised to RPS29 in both cell fractions from all non-diabetic donors (Figure 2C). As expected from the immunofluorescence experiments, high levels of insulin were seen in the beta cell fraction and glucagon in the alpha cell fraction. Insulin transcript in the alpha cells was low, as expected, but despite the small number of alpha cells seen in the beta cell fraction, glucagon transcript in the beta cells was unexpectedly high. There was no significant difference observed between male and female donors for either gene in either fraction (alpha cell glucagon had a tendency to be higher in RNA from male donors, but this did not achieve significance, p = 0.06).

### Insulin, glucagon and GLP-1 content measurement

Following our finding that glucagon mRNA was apparently highly expressed in the beta cells (Figure 2C) and in light of the discovery of GIP production in alpha cells by Fujita et al [30] we tested extracts of beta cells for insulin, glucagon and GLP-1 content. For the six sample sets in which insulin secretion and content had already been determined, we measured glucagon and GLP-1 content and compared these to the insulin content measurements (Figure 2D). The average concentrations of insulin, glucagon and GLP-1 were 39.9×10^3^ pg/ml, 1029 pg/ml and 31 pg/ml, respectively. Converted to molar concentrations, these values correspond to 7000 pM insulin, 294 pM glucagon and 9 pM GLP-1. The molar concentration of glucagon is therefore 4.2% of the molar concentration of insulin (p<0.05) in these samples, in striking agreement with the 4.73% average contamination of alpha cells in the beta cell fraction (Table 3). Our rationale for measuring GLP-1 was that beta cells express the prohormone convertase PC1/3, which is capable of cleaving preproglucagon peptide to generate GLP-1 [31]. However, GLP-1 content was at the limit of detection of the assay and at only 0.14% of the molar concentration of insulin in these beta cell extracts (p<0.05), showing that the beta cells are not synthesising this peptide either. We therefore conclude that the glucagon content is due to the cross contamination by alpha cells, but that human beta cells are capable of glucagon mRNA transcription.

### Type 2 diabetes susceptibility gene profiling of sorted islet cell RNA

**Figure 3:**
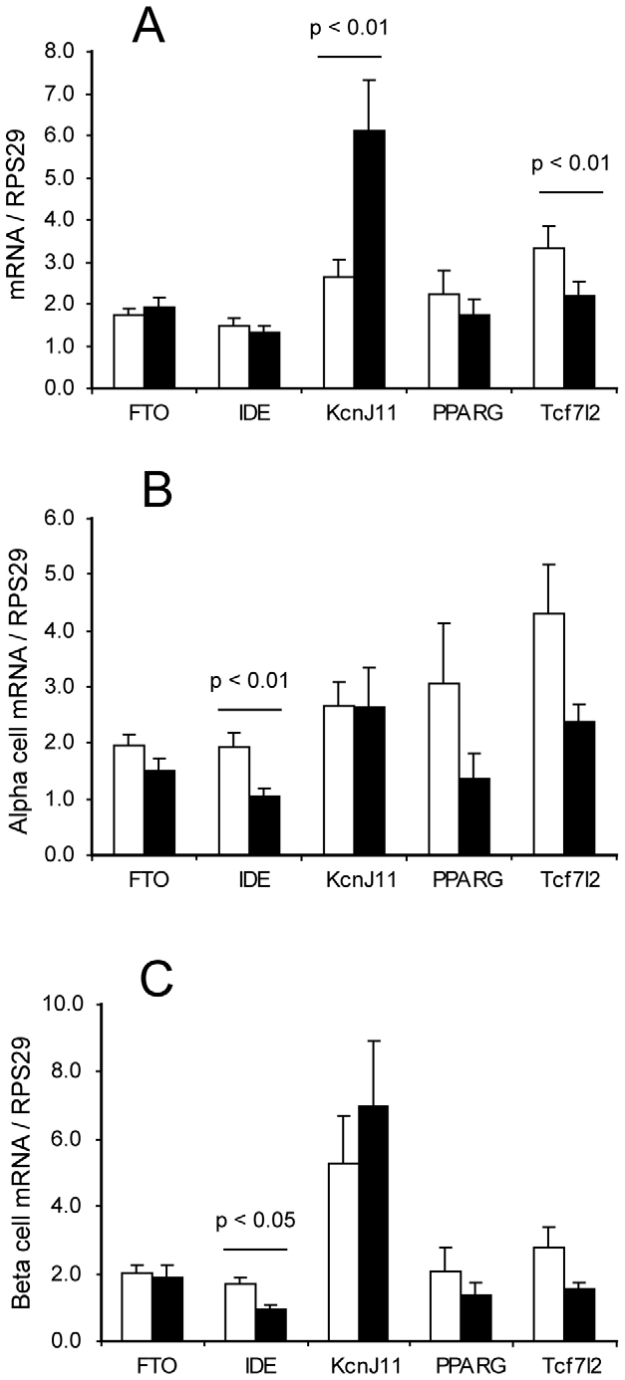
Type 2 diabetes susceptibility gene expression in sorted islet cells. A: mRNA expression of type 2 diabetes susceptibility genes in alpha cells (white bars) and beta cells (black bars), expressed as mean + SEM of all non-diabetic islet donors (n = 16). Statistical significance of pairwise comparisons between alpha and beta cell expression was by Student's T test assuming equal variance. B: Expression of the same data in alpha cells only with male (white bars) and female (black bars) islet donors considered separately (n = 8 each). C: Expression of the same data in beta cells only with male (white bars) and female (black bars) islet donors considered separately (n = 8 each).

We selected the genes *FTO*, *IDE*, *KCNJ11*, *PPARG* and *TCF7L2* as candidates to test for expression in the sorted islet cells. Transcript levels of these genes were measured in both cell fractions from all non-diabetic donors and normalised to RPS29. The ratio of expression (beta cells/alpha cells) was also calculated. All transcripts were observed in both alpha and beta cell fractions, and for *FTO*, *IDE* and *PPARG* there was no difference in the abundance of the transcripts between the alpha and beta cells. However, *KCNJ11* mRNA was enriched in the beta cells, while *TCF7L2* mRNA was enriched (although to a lesser extent) in the alpha cells (p<0.01 for both genes, Figure 3A). The data were analysed for sex-specific differences in the amounts of transcripts present in both cell fractions, as for insulin and glucagon, and the only difference observed was for *IDE* which was more abundant in male than in female cells for both cell types (Figure 3B and C, p<0.01 for the alpha cells and p<0.05 for the beta cells).

### Correlation of insulin gene expression with age

Multivariate ANOVA was performed to test for any interaction effects between age, BMI and sex of the islet donors on gene expression, in both cell types and on the ratio of expression in beta to alpha cells. No interaction effects were observed in two-way (age vs. BMI, BMI vs. sex and age vs. sex) or three-way (age vs. BMI vs. sex) comparisons. We also confirmed that average age and BMI of the islet donors was not significantly different between the sexes (Table 1) and that they did not correlate with each other (r = −0.195, p = 0.468). Having established that each of these variables could be considered separately, we calculated Spearman non-parametric correlation coefficients for correlations between insulin and glucagon mRNA expression and age or BMI of the islet donors, in both cell types. No association was observed between glucagon (in alpha or beta cells) and age or BMI. In the beta cells there was a slight negative association of insulin mRNA with donor age, although this did not reach significance after multiple testing correction (p = 0.056 after FDR).

### Correlations of type 2 diabetes susceptibility gene expression with age or BMI

**Figure 4:**
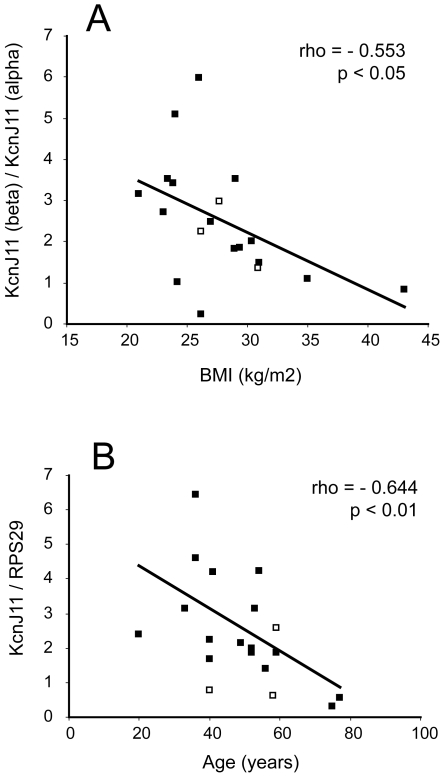
Correlations of type 2 diabetes susceptibility gene expression with age or BMI. Filled square data points represent non-diabetic donors, empty squares represent diabetic donors. Trendlines, Spearman correlation coefficients (rho) and their corresponding p values are calculated on the basis of the non-diabetic data points only. A: Correlation of the ratio of enrichment of *KCNJ11* mRNA in beta cells relative to alpha cells with BMI. B: Correlation of alpha cell *KCNJ11* mRNA with age.

**Figure 5:**
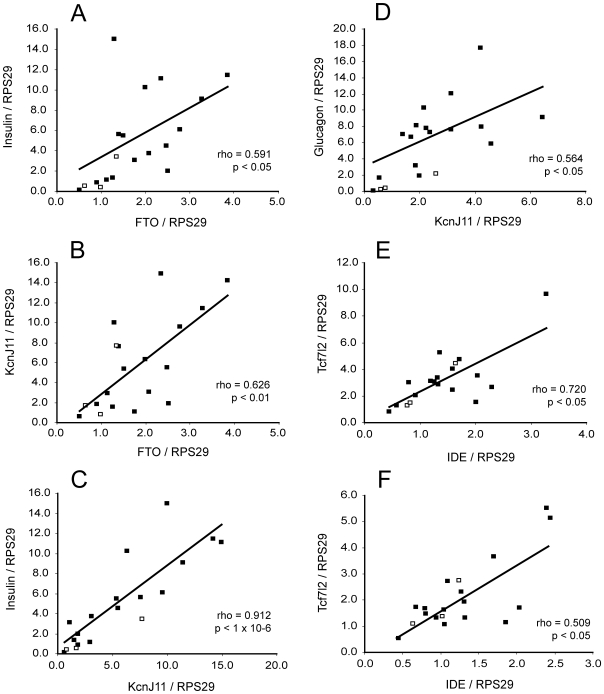
Correlations of mRNA levels with each other. Filled square data points represent non-diabetic donors, empty squares represent diabetic donors. Trendlines, Spearman correlation coefficients (rho) and their corresponding p values are calculated on the basis of the non-diabetic data points only. A: Correlation of beta cell insulin and *FTO*. B: Correlation of beta cell *KCNJ11* and *FTO*. C: Correlation of beta cell insulin and *KCNJ11*. D: Correlation of alpha cell glucagon and *KCNJ11*. E: Correlation of alpha cell *TCF7L2* and *IDE*. F: Correlation of beta cell *TCF7L2* and *IDE*.

We calculated correlation coefficients for *FTO*, *IDE*, *KCNJ11*, *PPARG* and *TCF7L2* mRNA expression with age or BMI in both cell types and for the ratio of expression in beta to alpha cells, for all samples together. No significant correlations with age or BMI were observed for *IDE*, *PPARG* or *TCF7L2*. However, the ratio of *KCNJ11* expression in beta to alpha cells for each donor was negatively correlated with BMI, suggesting that the enrichment of *KCNJ11* in the beta cells relative to the alpha cells is more pronounced in donors with low BMI and decreased in donors with high BMI (Figure 4A). No other correlation of gene expression with BMI was observed. We observed that *FTO* expression in beta cells had a slight negative correlation with donor age (although not quite at the level of significance after multiple testing correction, p = 0.067 after FDR), but no association with BMI. In the alpha cells, we also observed a negative correlation of *KCNJ11* transcript with age (Figure 4B). We calculated pairwise correlation coefficients for all the transcripts against each other, which revealed that in beta cells insulin, *KCNJ11* and *FTO* correlated positively with each other (Figure 5A, B and C). There was also a positive association between *KCNJ11* and glucagon in the alpha cells (Figure 5D), and between *TCF7L2* and *IDE* in both cell types (Figure 5E, alpha, and F, beta).

### Controlling for time spent in culture

**Figure 6:**
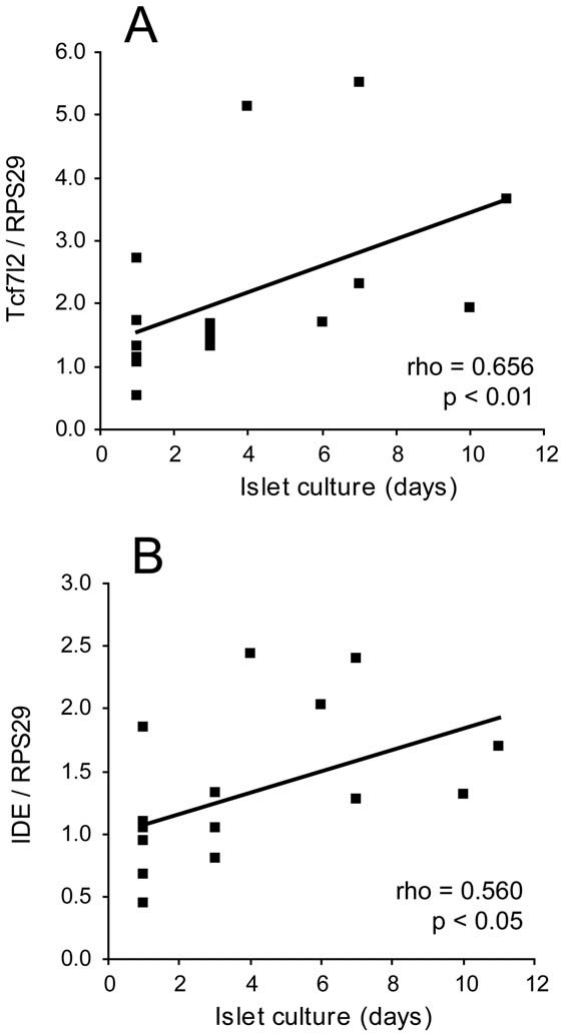
Effect of islet culture time on *TCF7L2* and *IDE* mRNA expression in non-diabetic islets. A: Scatter plot of beta cell *TCF7L2* mRNA against islet culture time prior to cell sorting. B: Scatter plot of beta cell *IDE* mRNA against islet culture time prior to cell sorting.

**Figure 7:**
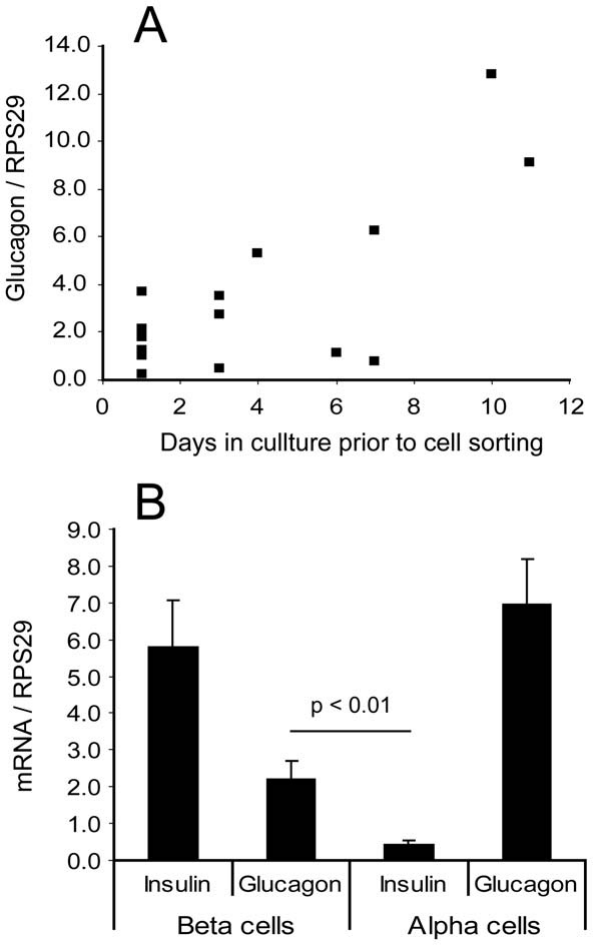
Effect of islet culture time on beta cell glucagon transcript levels. A: Scatter plot of beta cell glucagon mRNA against islet culture time prior to cell sorting (non-diabetic islets only). B: Average insulin and glucagon levels in non-diabetic islets excluding those which were cultured for 10 or 11 days (n = 14).

Since the islet preparations had not all spent the same length of time in culture, we investigated whether the culture time had affected transcription of any of the genes. Calculation of correlation coefficients between mRNA expression and number of days spent in culture showed that there was a positive association between culture time and expression of both *TCF7L2* and *IDE* in beta cells (Figure 6). In alpha cells, no correlation between culture time and gene expression was observed. For beta cell glucagon, although the correlation was not significant (p = 0.096), the two samples which had been cultured for 10 or 11 days prior to cell sorting had higher levels of glucagon transcript in the beta cells than the remainder of the samples which had been cultured for 7 days or less (Figure 7A). The high levels of glucagon in these two samples did not account for the average beta cell glucagon transcript being higher than the average alpha cell insulin transcript, as there was still significantly more beta cell glucagon than alpha cell insulin when the outlier values were excluded (Figure 7B, p<0.01 with or without the outliers). This finding suggests that the beta cells could dedifferentiate during islet culture with concomitant production of glucagon, although some non-alpha cell glucagon transcript is already present even in recently isolated islets.

### Type 2 diabetes susceptibility gene expression in sorted cells from diabetic islets

**Figure 8:**
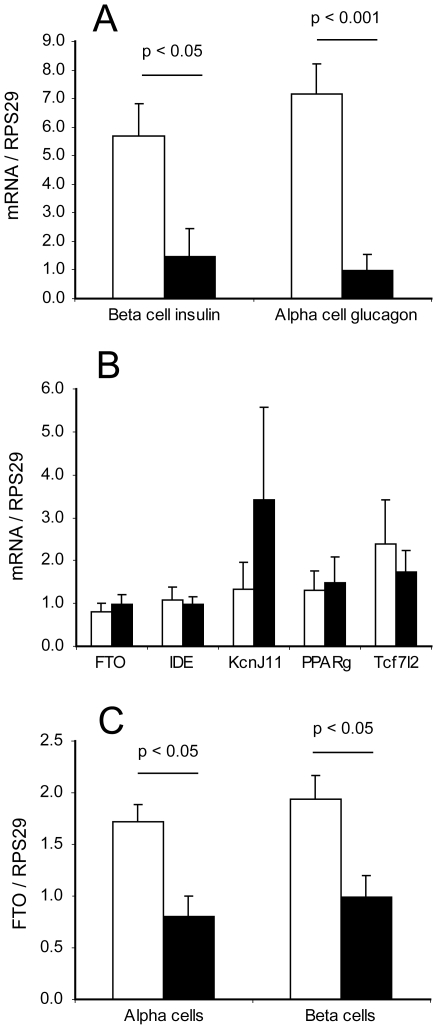
Insulin, glucagon and type 2 diabetes susceptibility gene expression in diabetic sorted islet cells. A: Differential expression of beta cell insulin and alpha cell glucagon in non-diabetic (white bars) compared to diabetic (black bars) islet cells. B: Expression levels of type 2 diabetes susceptibility gene in diabetic alpha cells (white bars) and beta cells (black bars). C: Differential expression of *FTO* in both cell fractions from non-diabetic (white bars) compared to diabetic (black bars) islet cells. Statistical comparison of diabetic to non-diabetic samples was by unpaired T test assuming unequal variance. n = 16 non-diabetic and 3 diabetic samples.

Alpha and beta cells were sorted from three preparations of type 2 diabetic islets and gene expression analysed as for the non-diabetic donors. Beta cell insulin and alpha cell glucagon were significantly lower in the diabetic cells than in the non-diabetic cells (Figure 8A, p<0.05 for beta cell insulin and p<0.001 for alpha cell glucagon). However, no difference was observed in beta cell glucagon transcripts between diabetic and non-diabetic cells. There was a tendency for glucagon to be proportionally higher relative to insulin in the diabetic beta cells (insulin/glucagon ratio was 5.53±2.41 in non-diabetic beta cells but 0.94±0.36 in diabetic beta cells), but this was not significant due to the variation in the non-diabetic cells (p = 0.078). Expression of the type 2 diabetes susceptibility genes in diabetic cells was similar (in amount and in *KCNJ11* beta cell enrichment) to their expression in non-diabetic cells (Figure 8B). However, significantly lower levels of *FTO* mRNA were observed in both alpha and beta cells from diabetic islets (Figure 8C, p<0.05 for alpha and beta cells, diabetic versus non-diabetic). The individual values were compared to those of the non-diabetic donors for each of the correlations previously established, and they did not noticeably deviate from the trendlines except for low levels of *KCNJ11* in the alpha cells (Figures 4D and 5D).

## Discussion

In this study we have examined expression of type 2 diabetes susceptibility genes in sorted islet cells in order to identify expression patterns associated with cell type or with phenotypic characteristics of the islet donors. We have found that the potassium channel subunit *KCNJ11* is enriched in human beta cells relative to alpha cells. This was surprising, given our previous finding that in rat islets this gene is more highly expressed in the alpha cells [21], and emphasises the importance of not assuming that rodent models can completely recapitulate the situation in human tissue. The negative correlation between the beta cell enrichment of *KCNJ11* and the BMI of the islet donors is suggestive of a link between BMI and islet function. However, there is no way of determining whether increased BMI is a cause or consequence of lower *KCNJ11* transcription in the beta cells. For example, it could be that increased fat mass puts more stress on the beta cell leading to decreased transcription of genes important for beta cell functionality, or alternatively that lower levels of *KCNJ11* mRNA in the beta cell relative to the alpha cell may result in changed insulin/glucagon ratio and hyperglycaemia, leading eventually to increased lipid production by the liver and enhanced fat deposition in the adipose tissue. Although we cannot distinguish between these scenarios, this work suggests a novel connection between the ATP sensitive potassium channel and obesity.

It was also surprising, given the literature on *FTO* expression in other tissue types, that we found no association between expression of *FTO* and BMI in islet cells. The mechanism of action of *FTO in vivo* is yet to be confirmed, although it has been shown to function *in vitro* as a DNA demethylase [32] and a transgenic mouse carrying a point mutation which decreases demethylase activity of *FTO in vitro* is protected from high fat induced weight gain [33]. Expression of *FTO*, regardless of SNP status, was found to be increased in adipose tissue from obese individuals relative to those of normal weight [11]. However, we only observed a slight association with age. This is in agreement with Grunnet *et al*, who recently found an age-dependent decline in *FTO* expression (in addition to associations with BMI and sex, depending on tissue type) in adipose tissue and skeletal muscle [34]. This study also reported correlations between *FTO* mRNA expression and the mRNA expression of various other functional genes, including *SLC2A4* (previously *GLUT4*), *PPARGC1A* and various genes involved in oxidative phosphorylation. This finding has interesting parallels with the correlation found by Zabena *et al* between *FTO* and adipokine expression in adipose tissue, and our findings that *FTO*, *KCNJ11* and insulin are positively correlated with each other in human beta cells. We could envisage that *FTO* might be a master regulator of metabolic function in various tissues and that an age-dependent decline in *FTO* expression is involved in the decrease of metabolic function occurring during the aging process. The association between *TCF7L2* and *IDE* suggests that *TCF7L2* may play a role in control of transcription of *IDE*. If confirmed, this would suggest a novel way by which *TCF7L2* contributes to beta cell viability, since *IDE* contributes to clearance of islet amyloid as well as insulin [23]. Intriguingly, on examination of the promoter of the *IDE* gene (NCBI accession no. NG_013012) we observed two sites with the sequence TGCAAAG at -756 and +240 bases relative to the transcription start site of *IDE*. This sequence is known to be a TCF binding site, and it is required for *TCF7L2*-mediated regulation of cyclin D1 transcription in human colon cancer cells [35]. A recent study found that *PPARG* could induce transcription of *IDE* in primary neurons [36], but we did not find any association between *IDE* and *PPARG* in our islet cell investigations. Indeed, we did not observe any difference in *PPARG* expression between cell types or between diabetic and non-diabetic samples, nor any association with phenotypic characteristics of the islet donors, as expected for a gene which exerts its major effect in other tissues than the islet. The positive association between *TCF7L2*, *IDE* and islet culture time might suggest that the beta cells are adopting a more alpha-cell-like state during islet culture, especially since average alpha cell *TCF7L2* mRNA was found to be higher than average beta cell *TCF7L2* (Figure 3A). Differences in *TCF7L2* mRNA expression have also been observed between functionally heterogeneous populations of rat beta cells, with the lower-responding “immature” beta cells expressing higher levels of TCF7L2 than the higher-responding “mature” population [37].

A surprising finding of this work was that the beta cell fraction displayed disproportionally high glucagon transcript relative to glucagon protein. Since the glucagon protein could be attributed to cross-contamination by alpha cells (4–5%), we conclude that the glucagon mRNA is produced, but is under post-transcriptional control, in the beta cells. This result is consistent with the findings of Katsuta et al [38] who discovered that insulin and glucagon are co-expressed in a subset of mature mouse beta cells. The mechanism of post-transcriptional regulation is unclear, but could involve islet cell-specific microRNAs. In this context, it is noteworthy that in the miR375 knockout mouse, beta cell mass was decreased and alpha cell mass increased, and the mice displayed hyperglucagonaemia [39]. It may be speculated that miR375 normally suppresses glucagon translation in the beta cell, which would be consistent with the phenotype of these mice. While islet culture time did not show a significant correlation with beta cell glucagon mRNA, it did appear to increase it after 7 days of islet culture. Increased beta cell glucagon after long periods of islet culture is in agreement with Russ *et al*, who found that labeled adult human beta cells started to express glucagon protein during dedifferentiation [40]. However, despite the presence of the glucagon transcript in the beta cells, during examination of the immunofluorescence images no double (insulin and glucagon) positive cell was ever observed. This is also in agreement with Katsuta *et al*, who found co-transcription of islet hormones in sorted mouse beta cells, but no colocalisation of immunofluorescence staining for the corresponding proteins [38]. It is still possible that other factors apart from islet culture time (such as cause of death of the donor, or cold ischaemia time) could affect gene expression in the sorted cells, but in culturing the islets for at least 24 hours we hoped to normalise the environment of the islets in order to minimise the effects of such external factors.

Although it was not unexpected to find lower levels of insulin in the diabetic beta cells, it was surprising to find decreased alpha cell glucagon, especially since one of the hallmarks of diabetes is inappropriately increased glucagon secretion [41]. However, we have only examined mRNA expression, which may not reflect glucagon secretion or content. The higher proportion of alpha cells observed in diabetic islets by Deng *et al* [42] may play a more important role in inappropriate glucagon release. However, this study also revealed lower maximal glucagon release during perifusion of diabetic islets when stimulated with KCl, possibly reflecting lower islet glucagon content. The decreased amount of *FTO* observed in both cell types from the diabetic islets is also intriguing. In a previous study comparing gene expression in diabetic islets against normal islets using DNA microarrays, *FTO* was not identified as a differentially regulated gene [43]. Further studies on the function of *FTO* in islets would be required to elucidate whether it plays a role in beta cell viability or dysfunction, and if so, how.

We acknowledge that this study is limited by a small number of samples, since the starting material can only be obtained from organ donors as opposed to peripheral tissue biopsies. Diabetic islets are less numerous and even more sensitive to the stress of the isolation process than normal islets ([42] and our own observations), and it is not often that enough diabetic islets can be obtained to perform the cell sorting procedure on them. In addition, we cannot account for any effects of SNPs on gene expression since the organ donors are not genotyped. This may explain why we were unable to reproduce the correlation between insulin and *TCF7L2* mRNA expression observed by Lyssenko *et al* in whole islets [12]. The primers used for *TCF7L2* in our study are located in exon 5, and would therefore have amplified all splice variants of *TCF7L2* mRNA [16], so we would have been unable to observe isoform-specific correlations. In the case of *FTO* it has been reported that there was no association between SNPs and *FTO* mRNA expression by both studies which have examined this parameter [11], [34] so our data on *FTO* should not be affected by not knowing the donors SNP status. We consider that our analysis of RNA expression of type 2 diabetes susceptibility genes in sorted alpha and beta cells has revealed interesting patterns of gene expression between cell types and between diabetic and non-diabetic samples, which may underpin future functional studies on the mechanisms of action of type 2 diabetes susceptibility genes.
